# The respiratory virome and exacerbations in patients with chronic obstructive pulmonary disease

**DOI:** 10.1371/journal.pone.0223952

**Published:** 2019-10-24

**Authors:** Anneloes L. van Rijn, Sander van Boheemen, Igor Sidorov, Ellen C. Carbo, Nikos Pappas, Hailiang Mei, Mariet Feltkamp, Marianne Aanerud, Per Bakke, Eric C. J. Claas, Tomas M. Eagan, Pieter S. Hiemstra, Aloys C. M. Kroes, Jutte J. C. de Vries

**Affiliations:** 1 Department of Medical Microbiology, Leiden University Medical Center, Leiden, the Netherlands; 2 Sequencing Analysis Support Core, Department of Medical Data Sciences, Leiden University Medical Center, Leiden, the Netherlands; 3 Department of Thoracic Medicine, Haukeland University Hospital, Bergen, Norway; 4 Department of Clinical Science, University of Bergen, Bergen, Norway; 5 Department of Pulmonology, Leiden University Medical Center, Leiden, the Netherlands; Imperial College London, UNITED KINGDOM

## Abstract

**Introduction:**

Exacerbations are major contributors to morbidity and mortality in patients with chronic obstructive pulmonary disease (COPD), and respiratory bacterial and viral infections are an important trigger. However, using conventional diagnostic techniques, a causative agent is not always found. Metagenomic next-generation sequencing (mNGS) allows analysis of the complete virome, but has not yet been applied in COPD exacerbations.

**Objectives:**

To study the respiratory virome in nasopharyngeal samples during COPD exacerbations using mNGS.

**Study design:**

88 nasopharyngeal swabs from 63 patients from the Bergen COPD Exacerbation Study (2006–2010) were analysed by mNGS and in-house qPCR for respiratory viruses. Both DNA and RNA were sequenced simultaneously using an Illumina library preparation protocol with in-house adaptations.

**Results:**

By mNGS, 24/88 samples tested positive. Sensitivity and specificity, as compared with PCR, were 96% and 98% for diagnostic targets (23/24 and 1093/1120, respectively). Additional viral pathogens detected by mNGS were herpes simplex virus type 1 and coronavirus OC43. A positive correlation was found between Cq value and mNGS viral normalized species reads (log value) (p = 0.002). Patients with viral pathogens had lower percentages of bacteriophages (p<0.001). No correlation was found between viral reads and clinical markers.

**Conclusions:**

The mNGS protocol used was highly sensitive and specific for semi-quantitative detection of respiratory viruses. Excellent negative predictive value implicates the power of mNGS to exclude any pathogenic respiratory viral infectious cause in one test, with consequences for clinical decision making. Reduced abundance of bacteriophages in COPD patients with viral pathogens implicates skewing of the virome during infection, with potential consequences for the bacterial populations, during infection.

## Introduction

Chronic obstructive pulmonary disease (COPD) is characterized by exacerbations with high morbidity and mortality, and over 65 million patients suffer from this disease worldwide [1]. A COPD exacerbation is an acute event leading to worsening of the respiratory symptoms and is associated with a deterioration of lung function [2]. Exacerbations are mainly associated with infections, of which a large part is caused by viruses (22–64%) [3–6]. However, in part of the exacerbations an etiologic agent is not detected.

Current routine virus diagnostics is based on polymerase chain reactions (PCR) and inherently the number of detectable pathogens is restricted to the ones included in the assay. Rare, mutated and pathogens with an uncommon clinical presentation will be missed, along with new and currently unknown ones. Over the last decades, several previously unidentified viruses have been discovered as respiratory pathogens, including metapneumovirus [7], middle-east respiratory syndrome coronavirus [8] and human bocavirus [9].

Metagenomic next generation sequencing (mNGS) is an innovative method, which enables the detection of all genomes in a given sample. Proof of principle studies have shown that mNGS on respiratory samples can confirm and extend PCR results and deliver typing and resistance data at the same time [10–14]. The performance of mNGS in the clinical diagnostic setting, especially the positive and negative predictive value, has not yet been elucidated and is likely to differ per clinical syndrome and sample.

Previous data from reports on 16S rRNA analysis from the respiratory tract have led to increased insight in the microbiome in patients with COPD [15]. Changes in bacterial populations have been associated with exacerbation events and clinical phenotypes [15]. However, these studies are intrinsically limited to analysis of the bacterial part of the microbiome.

So far only a few studies using shotgun metagenomics have focussed on the respiratory virome in children with acute respiratory infections [16, 17]. In this study, we analyse the composition of the virome in adult patients with exacerbations of COPD.

### Objectives

The aim of this study was to correlate the respiratory virome in COPD patients as found by mNGS with qPCR and clinical data.

## Materials and methods

### Patients

Patients with COPD were included in the Bergen COPD exacerbation study (BCES) between 2006 and 2010 in Bergen, Norway [18]. All patients lived in the Haukeland University Hospital district. Baseline data taken during the first visit while in the stable state included amongst others exacerbation history, medications, comorbidities, spirometry and Global Initiative for Chronic Obstructive Lung Disease (GOLD 2007) categorisation. Patients were given a telephone number to a study nurse, whom they would contact in case of an exacerbation. Patients with an exacerbation according to a predefined set of symptoms were scheduled for an appointment with a study physician the next working day. During exacerbations, nasopharynx swabs were sampled and two different markers for the severity of the exacerbation were scored. After an exacerbation a control visit was scheduled. During the study period 154 patients had at least one exacerbation and in total 325 exacerbations were included in BCES, of which 88 exacerbation samples were tested in the current study.

### Sample selection

Nasopharyngeal samples were frozen and stored at -80°C. In total 88 nasopharyngeal samples of patients at the time of exacerbation were selected based on the availability of other samples (outside the current focus) and sent to the Leiden University Medical Center (The Netherlands) for further testing.

### Lab-developed real-time PCR testing (qPCR)

The viral respiratory panel covered by the multiplex real-time PCR (qPCR) developed in our laboratory consists of coronavirus 229E, coronavirus HKU1, coronavirus NL63, coronavirus OC43, influenza A, influenza B, human metapneumovirus, parainfluenza 1–4 (differentiation with probes), respiratory syncytial virus, and rhinovirus [19].

Total nucleic acids (NA) were extracted directly from 200 μl clinical sample, using the Total Nucleic Acid extraction kit on the MagnaPure LC system (Roche Diagnostics, Almere, the Netherlands) with 100 μL output eluate. Nucleic acid amplification and detection by real-time PCR was performed on a BioRad CFX96 thermocycler, using primers, probes and conditions as described previously [19]. Cq values were normalized using a fixed baseline fluorescence threshold.

### Metagenomic next generation sequencing (mNGS)

The metagenomics protocol used has been described and optimized for simultaneously RNA and DNA detection previously [14]. In short, internal controls, Equine Arteritis virus (EAV) for RNA and Phocid Herpesvirus-1 (PhHV) for DNA (kindly provided by prof. dr. H.G.M. Niesters, the Netherlands), were spiked in 200 μl of the virus transport medium in which the nasopharyngeal swab was stored. Nucleic acids were extracted directly from 200 μl clinical sample using the Magnapure 96 DNA and Viral NA Small volume extraction kit on the MagnaPure 96 system (Roche Diagnostics, Almere, The Netherlands) with 100 μL output eluate (an updated version of the isolation method used for qPCR, tested previously [20]). Extraction buffer was used as negative control (for extraction, library preparation, and sequencing). For library preparation, 7 μl of nucleic acids were used, using the NEBNext^®^ Ultra^™^ Directional RNA Library Prep Kit for Illumina^®^, with several in-house adaptations to the manufacturers protocol in order to enable simultaneous detection of both DNA and RNA. The following steps were omitted: poly A mRNA capture isolation, rRNA depletion and DNase treatment step. This resulted in a single tube per sample throughout library preparation containing both DNA and RNA. Metagenomic sequencing was performed on an Illumina NextSeq 500 sequencing system (Illumina, San Diego, CA, USA), and approximately 10 million 150 bp paired-end reads per sample were obtained.

After quality pre-processing, sequencing reads were taxonomically classified with Centrifuge [21] using an index constructed from NCBI’s RefSeq and taxonomy databases (accessed February 2019) with reference nucleotide sequences for the viruses, bacteria, archaea, fungi, parasites, and protozoa. Reads with multiple best matches were uniquely assigned to the lowest common ancestor (k = 1 Centrifuge setting; previously validated [14]). Both negative and positive results were confirmed using GenomeDetective website [22] version 1.111 (accessed December 2018—January 2019) and horizontal coverage (%) was determined using GenomeDetective.

Read counts were normalized, dividing the rawread count by the total number of reads in the sample and by the (average) genome size, and multiplied by 10^11 (to achieve comprehensible read counts in the same order of magnitude as the raw read counts).

### Virus assembly

For samples with dubious or inconclusive classification results a *de novo* assembly was performed. Pre-processed short reads assigned to a higher taxonomic level of a suspected viral target were extracted and *de novo* assembled with SPAdes version 3.11.1 [23] into longer stretches of contiguous sequences (contigs). The resulting contigs were then run against the blast NCBI’s nucleotide (nt) database (accessed 2017) using blastn 2.7.1 [24]. After identification of a putative target sequence, all the reads from the original sample were mapped against the identified best BLAST hit for further confirmation using BWA 0.7.17 software package [25].

### Statistical analysis

Sensitivity, specificity, positive and negative predictive values were calculated based on 24 PCR positive and 1120 PCR negative target results of 88 samples.

Correlation between qPCR Cq value and logarithm of normalized numbers of mNGS viral reads was tested with population Pearson correlation coefficient.

Potential correlations of mNGS data with clinical variables were tested as follows. Cq value/ viral reads and clinical parameters (exacerbation severity, duration of exacerbation or decrease/increase in Forced Expiratory Volume in 1 second (FEV_1_, control visit compared to baseline) were tested with one-way ANOVA and Kruskal-Wallis test when appropriate (depending on distribution). Comparison of the percentage of phages of all viral reads (after subtraction of the internal control EAV and PhHV reads) between mNGS virus positive samples and negative samples was tested with Mann-Whitney U test, comparison with clinical parameters with Kruskal-Wallis test. Diversity of the virome in different patient groups was characterized by Shannon Diversity Index (H) and tested with Welch two sample t-test. Statistical analyses were performed using IBM SPSS Statistics version 25 software for Windows <0.05 were considered statistically significant.

### Ethical approval

Prior to inclusion all subjects received written and oral information and signed informed consent. The BCES study was approved by the regional ethical committee in Western Norway (REK-Vest, case- number 165.08). The performance of this study, including mNGS, was approved by the medical ethics review committee of the Leiden University Medical Center (CME number B16.004); no additional consent was necessary.

## Results

### Patients and samples

In total 63 patients with 88 exacerbations were included with a median of one exacerbation per patient (range 1–5). Baseline patient characteristics and exacerbation characteristics are shown in Tables 1 and 2 respectively.

**Table 1:** Baseline patient characteristics.

	Patients (n = 63)
Age median yrs (range)	63.5 (46.6–74.5)
Male sex	40 (64%)
BMI median, kg/m^2^ (range)	25 (15–39)
Body composition	
Cachectic	7 (11%)
Normal	24 (38%)
Overweight	22 (35%)
Obese	10 (16%)
Smoking	
Never	0 (0%)
Sometimes	37 (59%)
Daily	26 (41%)
GOLD stage	
II (FEV_1_ 50–80%)	29 (46%)
III (FEV_1_ 30–50%)	27 (43%)
IV (FEV_1_ <30%)	7 (11%)
FEV_1_ in % median (range)	0.49 (0.23–0.74)
>1 exacerbation past 12 months	16 (25%)
Inhalation steroids	50 (79%)

**Table 2:** COPD patient and exacerbation characteristics among patients having a viral or non-viral exacerbation.

	qPCR target virus		
	detected	not detected	
	n = 23	n = 65	P*
***Patient characteristics***			
*Sex*, *%*			0.21
Women	34.5	65.5	
Men	22.0	78.0	
*smoking status*, *%*			0.53
Ex-smoker	23.4	76.6	
Current-smoker	29.3	70.7	
*GOLD stage (2007)*, *%*			0.35
II (FEV_1_ 50–80%)	26.3	73.7	
III (FEV_1_ 30–50%)	30.8	69.2	
IV (FEV_1_ < 30%)	9.1	90.9	
*Frequent exacerbator*, *%*			0.72
No	25.0	75.0	
Yes	28.6	71.4	
*Using inhalation steroids*, *%*			0.55
No	20.0	80.0	
Yes	27.4	72.6	
*Age*, *mean yrs*	63.7	64.9	0.10
*BMI*, *mean kg/m*^*2*^	27.0	25.9	0.92
*FEV*_*1*_ *in % predicted*	49.3	47.5	0.48
***Exacerbation characteristics***			
*Exacerbation severity for entire exacerbation*			0.75
Mild (not requiring AB or oral steroids or hospitalization)	14.3	85.7	
Moderate (requiring AB or oral steroids)	26.9	73.1	
Severe (Emergency room or hospital admission)	28.6	71.4	
*Self-reported exacerbation severity at time of study sampling*			0.64
Dyspnea unchanged or increased on errands outside home	36.4	63.6	
Increased dyspnea doing housework	26.5	73.5	
Increased dyspnea at rest	28.6	71.4	
Must sit up at night due to dyspnea	14.3	85.7	
*CRP (ng/mL) at time of study sampling*†	32.5	34.2	0.27

*Pearson’s chi square test for categorical variables and t-test for continuous variables

^†^ missing data for 4 (1 virus positive, 3 virus negative) exacerbations

### Lab developed real-time PCR

Of the 88 samples, 23 (26%) tested positive with in-house PCR: 14 (61%) were rhinovirus positive, three influenza A, two coronavirus NL63, one coronavirus OC43, two parainfluenza 3 and one parainfluenza 4. Cq values ranged from 19–38 (Table 3).

**Table 3:** qPCR positive samples with respective mNGS results.

Samples	qPCR positive (%)	Cq values range	mNGS species positive (%)	mNGS species reads (range)	Coverage (%, range)
**All targets**	23/88 (26)	19–38	23/88 (26)	0–1,317,490	3–100
**Influenza A**	3/23 (13)	29–36	3/23 (13)	9–559	3–98
**Cov NL63**	2/23 (9)	32	2/23 (9)	1,347–127,284	93–100
**Cov OC43**	1/23 (4)	27	2*/23 (4)	72,644–1,317,490	99–99
**PIV3**	2/23 (9)	26–36	2/23 (9)	59–288,877	14–99
**PIV4**	1/23 (4)	24	1/23 (4)	185,235	100–100
**Rhinovirus**	14/23 (61)	19–38	13**/23 (57)RV-A: 6/13RV-B: 2/13RV-C: 5/13	0–310,491 32–27,096 13,445–18,206 217–310,491	94–100 100–100 30–100

*Retesting by qPCR confirmed the OC43 finding of mNGS

** Rhinovirus not detected with mNGS had PCR Cq value 38

### Metagenomic next generation sequencing

A median of 11 million (7,522,643–20,906,019) sequence reads per sample were obtained. Of the 11 million reads, approximately 93% were *Homo sapiens* reads, 3% were bacterial and 0.1% viral (Table 4). A median of 3% of the reads could not be assigned to sequences in the Centrifuge index database (NCBI RefSeq).

**Table 4:** mNGS read counts.

	Median	Min	Max
Total reads	10,764,981	7,522,643	20,906,019
% unassigned reads	3	0.7	22
Homo sapiens reads(% total)	9,470,904 (93)	2,491,763	18,646,521
Bacterial reads (% total)	285,567 (3)	6,289	10,490,131
Viral reads (% total)	15,679 (0.1)	803	1,553,567
**PhHV reads (% viral)**	3,289 (22)	299	26,623
**EAV reads (% viral)**	10,152 (72)	197	75,771

### Comparison of mNGS to qPCR

**Fig 1:**
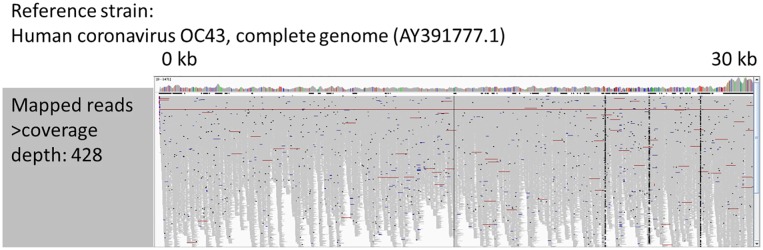
Coverage plot of betacoronavirus reads to coronavirus OC43 reference genome AY391777.1 (depth of coverage: 428).

Of the 23 qPCR positive samples, 22 tested positive with mNGS, resulting in a sensitivity of mNGS of 96%. Only one sample, that was rhinovirus positive by qPCR (Cq 38), could not be detected by mNGS (Table 3). Coverage of reference genomes was high (93–100%) with the exception of three samples: 30% coverage of rhinovirus C (1,401,120 mapped reads, 88,353-fold depth), 14% coverage of parainfluenza 3 (50 mapped reads, 3-fold depth), and 3% coverage of influenza A virus (single genome segment, 8 mapped reads). Aligning reads with Bowtie confirmed the rhinovirus C and parainfluenza 3 mapping, but not the influenza A mapping. Additional viral pathogens detected by mNGS were herpes simplex virus type 1 (17,031 reads, 82% coverage, 36-fold depth) which was not in qPCR viral respiratory panel (retrospectively confirmed by means of in-house HSV PCR; Cq 24), in the sample with the 8 influenza virus reads, and a betacoronavirus. Since coronaviruses tested negative by means of qPCR, and the mNGS classification was inconclusive, the reads were *de novo* assembled. Of these 83,252 betacoronavirus reads, *de novo* assembly resulted in 3 contigs (size 30743, 274 and 232 bp respectively) with best BLAST hit coronavirus OC43 (reference genome GenBank accession AY391777.1). A coverage plot of all reads against this reference strain (Fig 1) showed good horizontal and vertical coverage (read coverage depth 428). The original OC43 qPCR amplification appeared to have been inhibited, and repeated OC43 qPCR confirmed the positive mNGS result (Cq 25).

#### Sensitivity, specificity and predictive value

**Fig 2:**
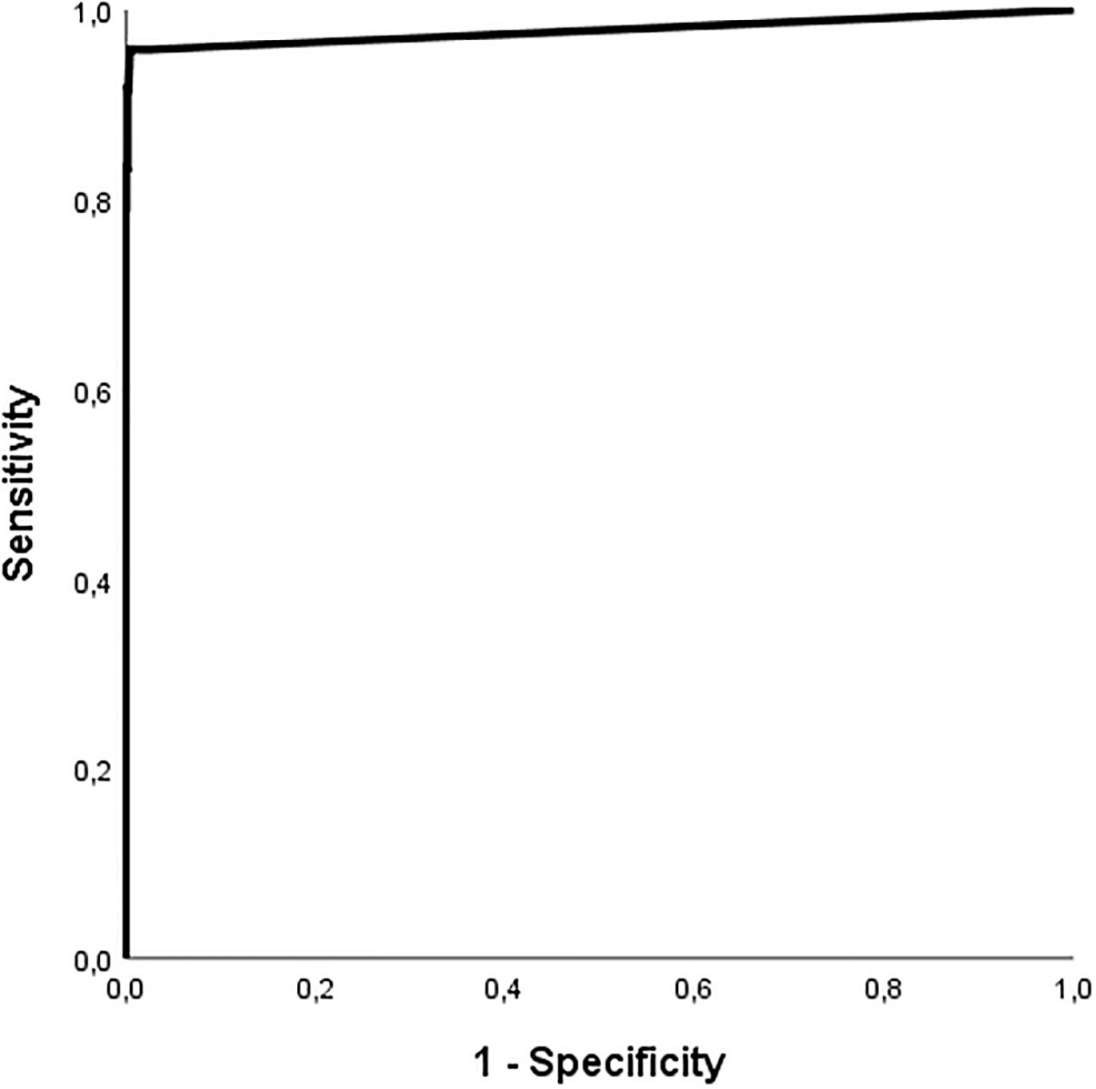
ROC curve of cut-off levels of mNGS normalized reads.

The sensitivity, specificity and predictive values of mNGS were calculated based on 24 PCR positive and 1120 PCR negative target results of 88 samples and the normalized read counts (Table 5). Calculations were made using different cut-off values of respectively ≥0, ≥15 and ≥50 normalized read counts. With a cut-off of ≥15, the sensitivity was 92% and specificity 100% and the positive predictive value (PPV) increased to 92%. The negative predictive value (NPV) was 100% for all cut-off levels. A ROC curve (Fig 2), using Youden’s index [26] demonstrated that the optimal sensitivity and specificity were achieved using a cut-off of 5 reads (96% (23/24) and 100% (1115/1120) respectively).

**Table 5:** Sensitivity and specificity of mNGS normalized reads for PCR target viruses. PPV: positive predictive value, NPV, Negative predictive value.

	Cut-off number of reads		
	0	15	50
Sensitivity	96% (23/24*)	92% (22/24)	83% (20/24)
Specificity	98% (1093/1120)	100% (1118/1120)	100% (1118/1120)
PPV	46% (23/50)	92% (22/24)	91% (20/22)
NPV	100% (1093/1094)	100% (1118/1120)	100% (1118/1122)

*The sample with positive confirmatory OC43 PCR included.

### Typing

mNGS provides additional typing data, as compared to qPCR. Of the 13 rhinoviruses detected with mNGS, 6 (46.2%) were rhinovirus A, 2 (15.4%) rhinovirus B and 5 (38.5%) rhinovirus C. The three influenza viruses were assigned to be H3N2 strains by mNGS.

### Semi-quantification by means of mNGS read count

**Fig 3:**
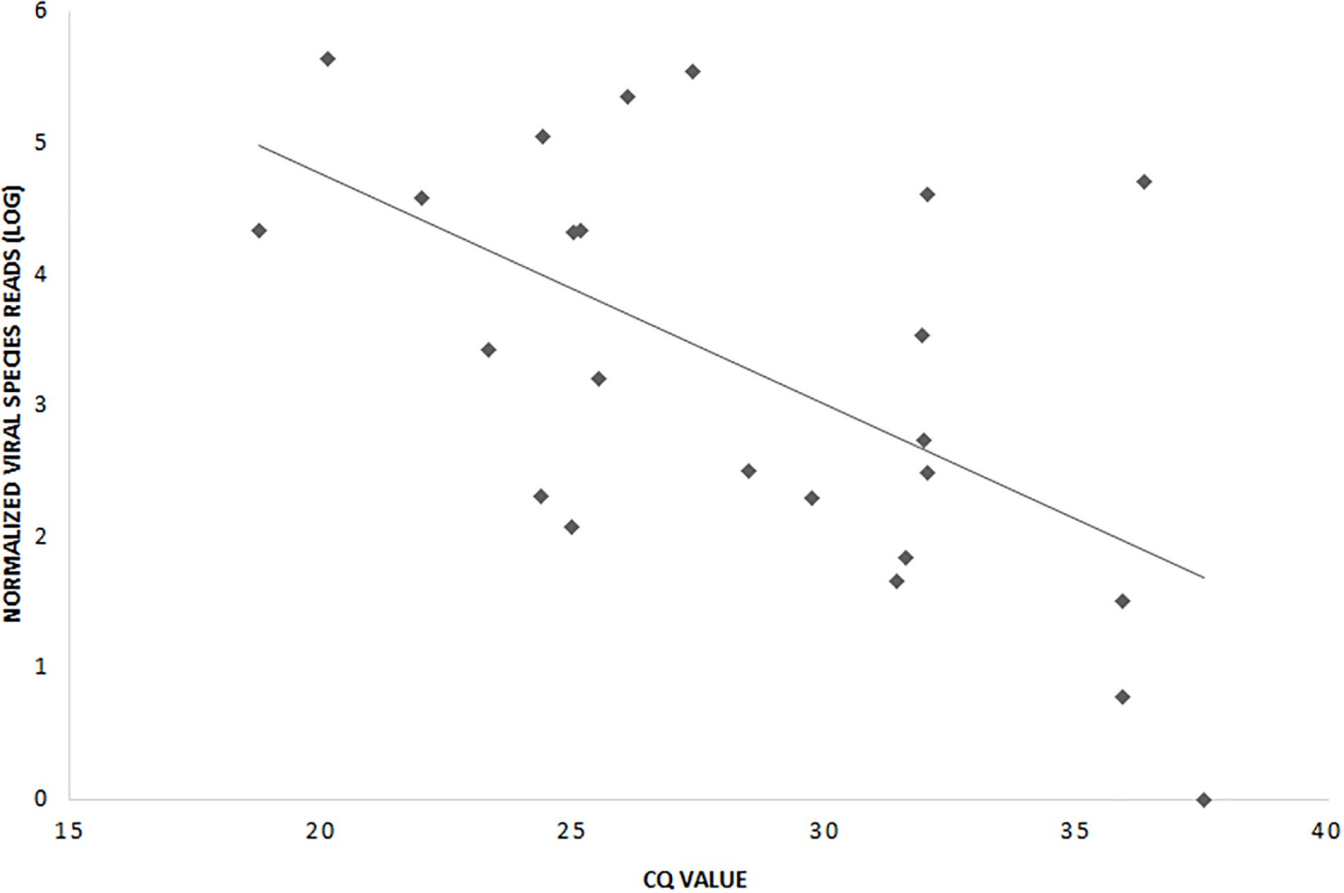
Correlation between mNGS normalized viral species reads (log) and Cq value (ρ = -0.6, p = 0.002).

In order to analyse the semi-quantitative quality of the mNGS assay, the number of the normalized sequence reads (log) mapping to qPCR target viruses (species level) as obtained with mNGS were compared to the Cq values of qPCR. A significant negative correlation was found (Fig 3; Pearson correlation coefficient ρ = -0.6, p = 0.002).

### Clinical parameters and mNGS pathogen read count

The following markers were tested for potential associations with clinical severity of exacerbation (exacerbation severity, self-reported exacerbation severity), length of exacerbation and a decrease/increase in FEV_1_ (control visit compared to baseline): mNGS pathogen positive versus negative exacerbation (qPCR targets), the number of normalized reads (log, cut-off of ≥5normalized reads) for the different target viruses (species level). No correlation was found between these markers and the different disease severity parameters (results not shown).

### The respiratory virome

**Fig 4:**
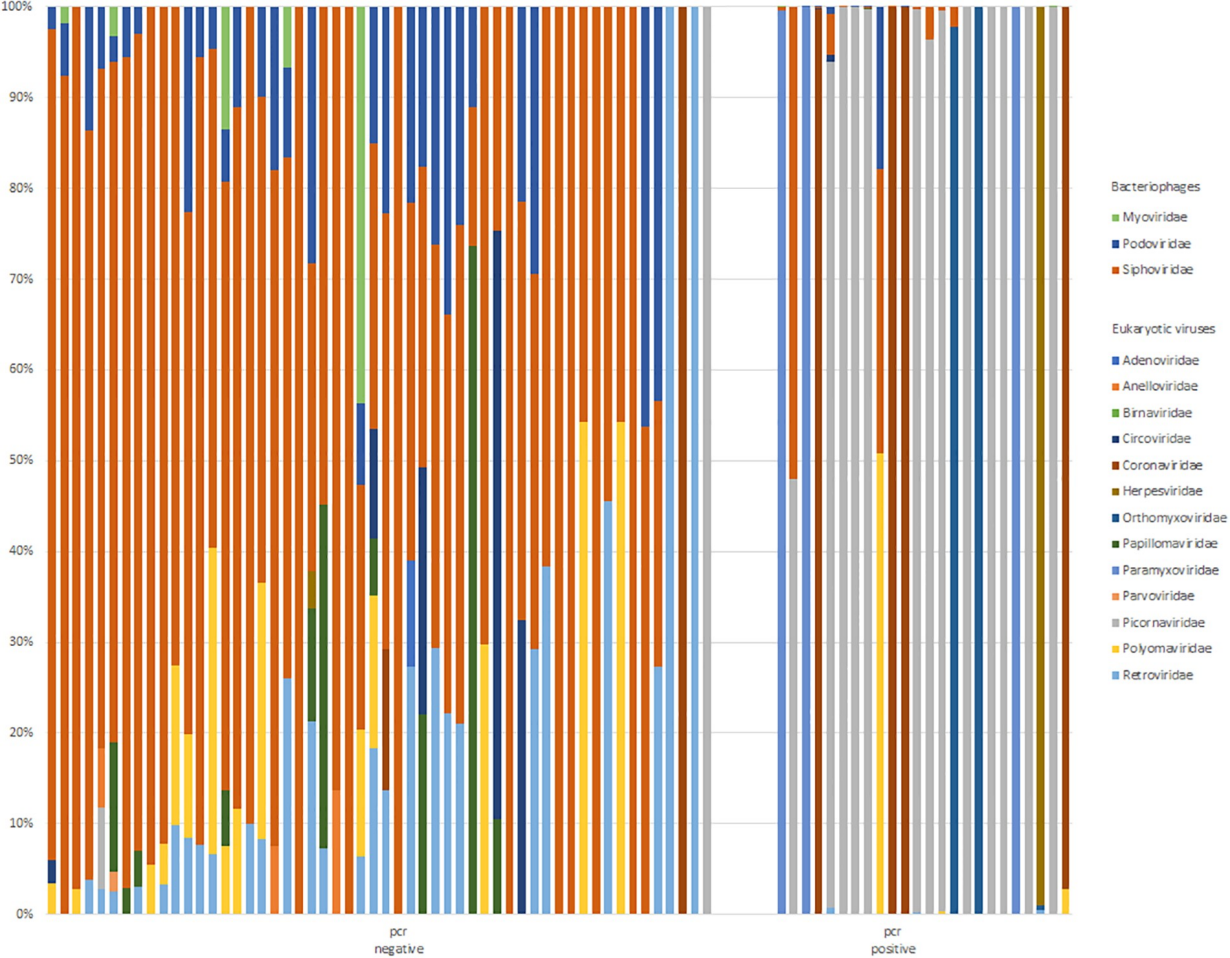
The respiratory virome: Proportion of normalized read counts of viral families per patient. Internal control reads (EAV and PhHV-1) excluded, ten patients without viral reads excluded.

**Fig 5:**
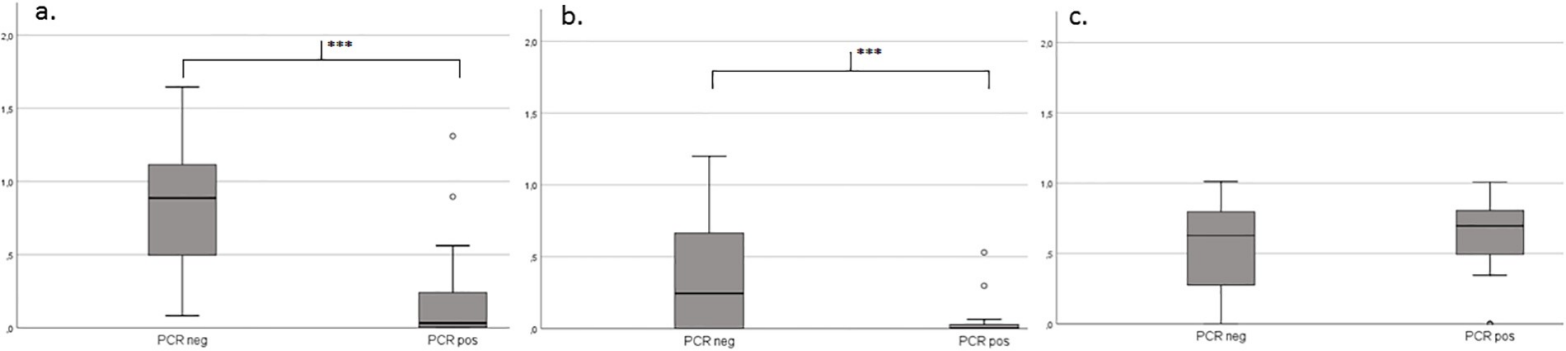
Shannon diversity scores for: (a) viruses, (b) eukaryotic viruses, (c) bacteriophages. COPD exacerbations of viral etiology had significant lower diversity (b). Boxes span IQR, *** significant (a) all viruses (P<0.001) and (b) eukaryotic viruses (p = 0.028), ○ outliers.

Overall proportions of normalized read counts of viral families (excluding EAV and PhHV control reads, cut-off of ≥5normalized reads) detected by mNGS per patient are shown in Fig 4. Patients with viral pathogens (PCR target viruses) had significantly reduced proportions of bacteriophages when compared to patients without viral pathogen:0% and 79% bacteriophages respectively (p<0.001) bacteriophage reads vs. all viral reads, normalized reads excluding EAV and PhHV control reads. The Shannon diversity scores for bacteriophages (normalized reads, cut-off of ≥5normalized reads) were comparable for COPD exacerbations of viral aetiology in PCR positive versus negative patients (Fig 5). Shannon diversity (normalized reads, excluding internal controls) was significant lower for all viral reads(P<0.001) and eukaryotic viruses (p = 0.028) in patients with viral pathogens (PCR target viruses positive).

No significant association was found between the diversity scores, nor the percentage of bacteriophages, and the following parameters: disease severity, length of exacerbation, number of exacerbations during the study period, difference in FEV_1_, GOLD stage, smoking, CRP level, and the virus species (results not shown).

### The respiratory bacteriome

**Fig 6:**
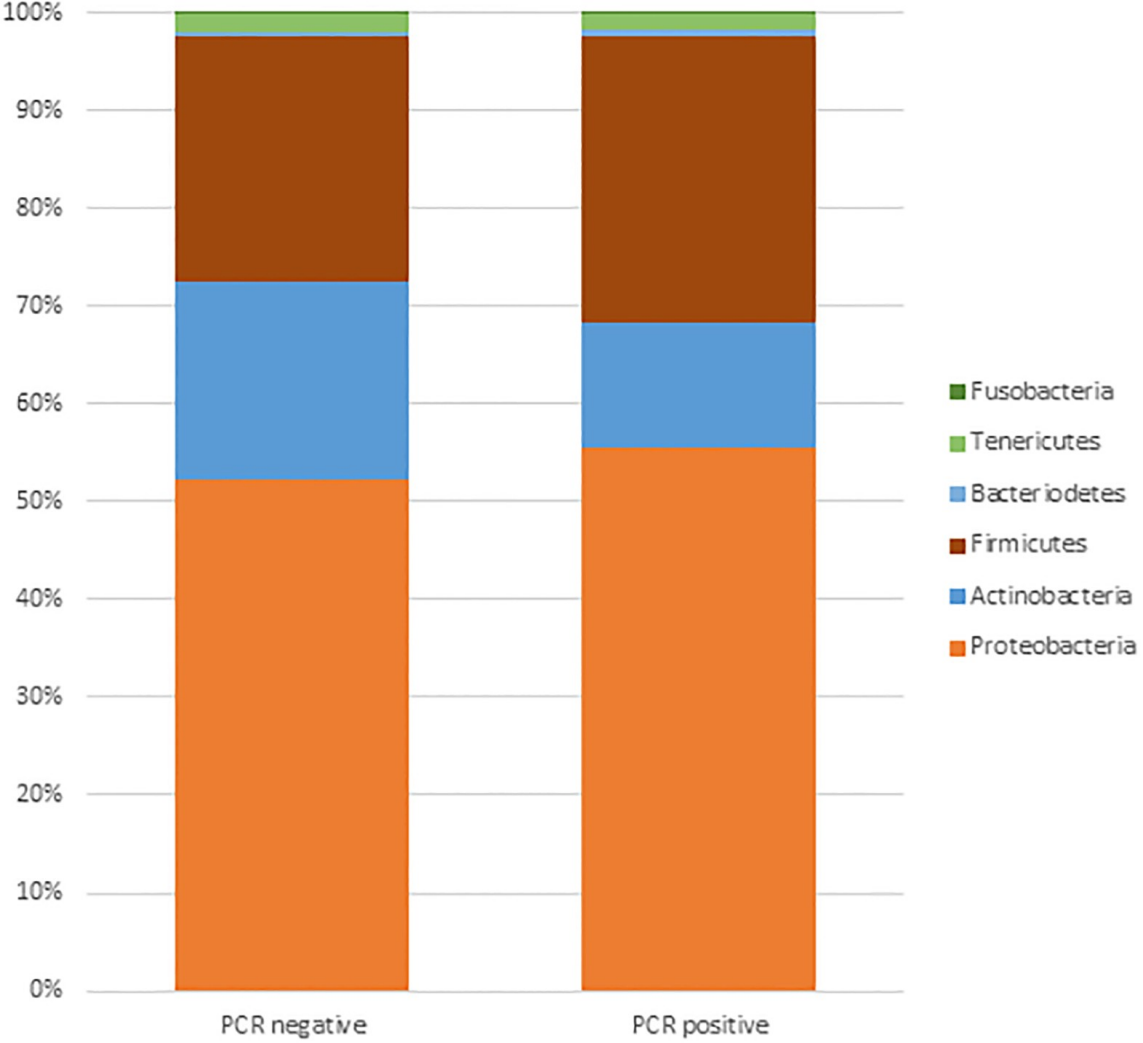
Proportion of normalized bacterial reads per phylum.

The most prevalent phyla were Proteobacteria, Firmicutes Actinobacteria and Bacteroidetes, see Fig 6.

The normalized bacterial read count of the most prevalent phyla was not significantly different between patients with a PCR-target virus positive and PCR-target negative patients.

Pathogenic bacterial species detected with an abundance of >10% of the bacterial reads were: *H*. *influenza (five samples)*; *M*. *catarrhalis (20 samples)*; *S*. *pneumoniae (one sample)*; and *S*. *aureus (one sample*). No apparent association with bacteriophages was found, or was a high abundance of bacteriophages associated with COPD exacerbations of viral cause.

### Data access

The raw sequence data of the samples, after removal of human reads have been deposited to Sequence Read Archive database (http://www.ncbi.nlm.nih.gov; accession number SRX6713943-SRX6714030).

## Discussion

In this study, the respiratory virome in patients with COPD exacerbations was analysed with both mNGS and qPCR, and combined with clinical data. The incidence of viral pathogens was 26% with both mNGS and qPCR. mNGS failed to detect one Rhinovirus with low load (Cq 38) and PCR failed to detect one betacoronavirus OC43 (72644 reads), due to one of the limitations of PCR, *i*.*e*. inhibition of amplification. One additional viral pathogen, not present in the respiratory PCR panel, was detected: herpes simplex virus 1, found by others to be associated with COPD [27].

The incidence of viral pathogens was comparable to that in previous publications (22–64%) [3, 5, 6]. The viral pathogen with the highest incidence was rhinovirus, followed by influenza, coronaviruses and para-influenza viruses. Interestingly, subtyping data was readily available by mNGS, accentuating the high resolution of mNGS, with rhinovirus (RV) species A and C being most frequent, followed by RV-B. RV-C was first identified in 2006 and associated with high symptom burdens in children and asthmatics [28, 29]. Recently, an asthma-related cadherin-related family member 3 (CDHR3) gene variant [30] was associated with greater RV-C receptor display on pulmonary cell surfaces of children and adults, and associated with higher susceptibility to severe virus-triggered asthma episodes [31, 32]. In line, Romero-Espinoza et al detected predominantly RV-C in children with acute asthma exacerbations by mNGS [33]. The significance of RV-C infection in the adult population is less well studied. Although RV-C has been previously associated with exacerbations of COPD [34, 35], to our knowledge, to date, CDHR3 polymorphisms have not yet found to be associated with COPD.

The sensitivity, specificity and positive and negative predictive values of mNGS were high: 96%, 100%, 82% and 100%, respectively, when encountering a cut-off of ≥5normalized reads, with a detection limit of approximately Cq 38. The high negative predictive value implicates the potential of mNGS to exclude the most prevalent viral respiratory infections in one test. The potential to exclude any infectious cause, both viral and bacterial, would have significant consequences for starting and/or continuation of antimicrobial or, at the other end of the spectrum, immune-modulating treatment.

The normalized viral species sequence read count might give an indication of the viral burden and the clinical relevancy of the detected virus. Although in our dataset we could not find any correlation with disease severity, several paediatric studies demonstrated a correlation between virus load and severity in respiratory infections [36–39]. Further analysis with a larger number of infected patients and/or a different spectrum of exacerbation severity will be needed to demonstrate or exclude such an association in COPD patients.

Furthermore, the complete respiratory virome showed a high bacteriophage abundance that could be linked to the absence of viral pathogens. Lower bacteriophage abundance may be the result of viral expansion. Hypothetically, a healthy virome size and diversity fits a certain size and diversity of bacteriophages, while during viral infection, pathogens predominate the virome. Alternatively, others have hypothesized that viral and microbial diversity may play a role in infection susceptibility and the development of acute and chronic respiratory diseases [33]. Our results indicate that virome dysbiosis may be accompanied by bacteriome dysbiosis, though no significant differences were detected in line with other reports [40, 41]. However, these studies don’t compare between COPD exacerbation with and without viral infections. Others have found a higher phage abundance in a patient with severe COPD when compared with one patients with moderate COPD and healthy controls, DNA sequencing, in line with the hypothesis of a state of dysbiosis that increases with disease progression [27]. In COPD patients, viral infections have been suggested to trigger bacterial overgrowth and infections [42, 43], demonstrating the significance of viral-bacterial interactions. Moreover, hypothetically, bacteriophages play a role in the horizontal gene transfer of bacterial virulence factors.

The most abundant bacterial phyla detected in this study were comparable with other reports. Although the percentage of proteobacteria was relatively high when compared to other studies of the nasopharyngeal microbiome, our swabs are sampled during COPD exacerbations [44, 45]. Study of the lower airways by means of e.g. protected brushes during bronchoscopy is needed for further analysis of bacterial and viral (sub)populations including comparison with PCR and culture results. Studies comparing the respiratory virome during stable disease and exacerbations are needed to determine a potential correlation between the virome/bacteriome during stable state and disease progression or exacerbation frequency.

In the current study, most respiratory pathogens detected were RNA viruses. This is in line with previous literature [3, 5, 6]. However, it must be noted that, despite the fact that a wide range of DNA viruses have been detected with the current protocol (DNA bacteriophages with high abundance, herpes simplex virus, bocavirus, anelloviruses, CMV, KI polyomavirus [14]), we cannot exclude suboptimal detection of some DNA viruses. Furthermore, highly divergent viruses with sequences deviating from their representative NCBI RefSeq sequences may have been missed, as has been described by others [46]. However, bioinformatic classification using alternative databases (both GenomeDetective and local databases) did not result in additional findings.

Though mNGS renders the possibility to detect all viruses in direct respiratory material, this revolutionary method is not yet used as routine accredited diagnostic procedure for pathogen detection. Before mNGS can be implemented as a routine diagnostics, the optimal protocol must be defined and analysis and interpretation of the metagenomic data must be standardized, followed by external quality assessment. This study demonstrates good performance of our mNGS protocol, in line with other studies [37, 38, 47, 48] and seems to overcome some of the current thresholds for implementation in clinical diagnostics.

## Conclusions

The mNGS protocol used was highly sensitive and specific for semi-quantitative detection of respiratory pathogenic viruses. Excellent negative predictive value implicates the potential of mNGS to exclude a known viral infectious cause in one test, with consequences for clinical decision making. Reduced abundance of bacteriophages in COPD patients with viral pathogens implicates skewing of the virome, and speculatively the bacterial population, during infection.
